# The Treatment of Scratches or Grease

**Published:** 1882-04

**Authors:** 


The Treatment of Scratches or Grease.—This disease consists of an inflammation of the heel of the horse, ending in fissures, ulcers, and fungous granulations. When very chronic a thick oily discharge is given off, from which the trouble has its name. It is not infrequent in this city, and coachmen impute it to the snow or snow and salt. It is not likely to occur, however, when the legs are kept properly cleaned.

The disease is not rare nor hard to diagnose. It is strange, therefore, that even among veterinarians the principles of treatment are yet but ill understood. Even in good text-books the old-fashioned treatment of wiping dry or washing and applying lard is still used.

In all except the early stages, the treatment should be to cleanse thoroughly and then apply astringents. One of the best is chloride of zinc in the proportion of gr. xxx to the pint of water. Glycerine may be added in order to make the astringent action more permanent.

